# Target organ damage and cardiovascular complications in patients with hypertension and type 2 diabetes in Spain: a cross-sectional study

**DOI:** 10.1186/1475-2840-5-23

**Published:** 2006-11-03

**Authors:** Luis Cea-Calvo, Pedro Conthe, Pablo Gómez-Fernández, Fernando de Alvaro, Cristina Fernández-Pérez, RICARHD investigators

**Affiliations:** 1 Clinic Research Department, Merck Sharp & Dohme, Madrid, Spain; 2 Internal Medicine Department, Hospital Gregorio Marañón, Madrid, Spain; 3 Nephrology Department, Hospital del SAS, Jerez, Cádiz, Spain; 4 Nephrology Department, Hospital La Paz, Madrid, Spain; 5 Clinical Research Support Department, Hospital Clínico Universitario San Carlos, Madrid, Spain; 6 See Acknowledgements for the complete list

## Abstract

**Background:**

Target organ damage (mainly cardiac and renal damage) is easy to evaluate in outpatient clinics and offers valuable information about patient's cardiovascular risk. The purpose of this study was to evaluate, using simple methods, the prevalence of cardiac and renal damage and its relationship to the presence of established cardiovascular disease (CVD), in patients with hypertension (HT) and type 2 diabetes mellitus (DM).

**Methods:**

The RICARHD study is a multicentre, cross-sectional study made by 293 investigators in Nephrology and Internal Medicine Spanish outpatient clinics, and included patients aged 55 years or more with HT and type 2 DM with more than six months of diagnosis. Demographic, clinical and biochemical data, and CVD were collected from the clinical records. Cardiac damage was defined by the presence of electrocardiographic left ventricular hypertrophy (ECG-LVH), and renal damage by a calculated glomerular filtration rate (GFR) of <60 ml/min/1.73 m^2^, and/or the presence of an albumin/creatinine ratio ≥ 30 mg/g; or an urinary albumin excretion (UAE) ≥ 30 mg/24 hours.

**Results:**

2339 patients (mean age 68.9 years, 48.2% females, 51.3% with established CVD) were included. ECG-LVH was present in 22.9% of the sample, GFR <60 ml/min/1.73 m^2 ^in 45.1%, and abnormal UAE in 58.7%. Compared with the reference patients (those without neither cardiac nor renal damage), patients with ECG-LVH alone (OR 2.20, [95%CI 1.43–3.38]), or kidney damage alone (OR 1.41, [1.13–1.75]) showed an increased prevalence of CVD. The presence of both ECG-LVH and renal damage was associated with the higher prevalence (OR 3.12, [2.33–4.19]). After stratifying by gender, this relationship was present for both, men and women.

**Conclusion:**

In patients with HT and type 2 DM, ECG-LVH or renal damage, evaluated using simple methods, are associated with an increased prevalence of established CVD. The simultaneous presence of both cardiac and renal damage was associated to the higher prevalence of CVD, affording complementary information. A systematic assessment of cardiac and renal damage complements the risk assessment of these patients with HT and type 2 DM.

## Background

The presence of diabetes mellitus (DM) increases the risk of any form of cardiovascular disease (CVD) and of death in hypertensive patients [1]. In the natural course of arterial hypertension (HT) it moreover has been seen that the development of type 2 DM during treatment multiplies the risk of cardiovascular complications over the middle term [2].

In the outpatient setting, the detection of silent cardiac damage (mainly left ventricular hypertrophy [LVH]) [3,4], or of renal disease (pathological urinary albumin excretion [UAE] [5,6] or diminished glomerular filtration rate [GFR] [7,8]) in patients with HT and/or DM, defines a subgroup in whom cardiovascular risk is even greater. The detection of such target organ damage is simple in daily clinical practice, based on the electrocardiogram (ECG) and assessment of kidney function and UAE. Specifically, in patients with HT and DM, this identifies patients at very high cardiovascular risk. In the LIFE study, on patients with HT and electrocardiographic left ventricular hypertrophy (ECG-LVH), mortality during a follow-up period of 5 years was 14% [9], figure that was even greater among patients with elevated UAE [10].

The implication of target organ damage in the appearance of cardiovascular complications, and the possibility of adopting treatments to induce regression of such damage – with improvements in patient prognosis in some cases –, make it necessary to carefully assess silent organ damage. Epidemiological studies conducted in our setting and involving hypertensive subjects have shown a prevalence of ECG-LVH of 10–20% [11-13], with a prevalence of kidney damage of 20–30% [14]. However, no studies to date have evaluated in Spain the prevalence of target organ damage based on simple methods (basically ECG and blood and urine tests), and its impact upon the prevalence of established CVD in patients with HT and type 2 DM. The main objective of the RICARHD study (Cardiovascular risk in patients with arterial hypertension and type 2 diabetes) was to evaluate the prevalence of hypertensive cardiac and renal damage using the methods commonly used in outpatient clinics, and its relationship to the presence of established CVD, in a population of patients with HT and type 2DM.

## Patients and methods

The RICARHD study was an epidemiological, multicentre, cross-sectional study conducted by 293 physicians specialized in Internal Medicine or Nephrology, in outpatient consulting offices. The study was approved by an independent Clinical Research Ethics Committee. The data collection period was between October and December 2005. Each investigator recorded information of 10 patients with HT and type 2 DM. In order to reduce selection bias, inclusion was requested of the first two or three programmed patients during 4–5 consecutive days. The study protocol was explained to the patients, and written informed consent was obtained.

The study comprised patients aged 55 years or older, with a diagnosis of HT and type 2 DM – both disorders having been present for more than 6 months. The presence of nephropathy not caused by DM or HT, and patient refusal to take part in the study were considered exclusion criteria. The clinical data were obtained from the patient history, while the biochemical parameters were recorded from laboratory testing in the three months prior to consultation (or in the days after consultation if no such prior testing proved available). Blood pressure (BP) recordings were made twice, under baseline conditions, and spaced one minute apart. Patient smoking or the consumption of coffee or other stimulants was not allowed before these measurements were obtained.

### Evaluation of the main objective

The main objective of the study was to evaluate the prevalence of cardiac [LVH] and renal damage, based on the ECG and laboratory tests, in patients diagnosed with HT and type 2DM, and its relationship to the presence of established CVD.

ECG-LVH was diagnosed based on the voltage criteria of Cornell [15], and of Sokolow-Lyon [16]. The presence of ECG-LVH was accepted if the patient met: a) the voltage criterion of Cornell (sum of the R-wave on lead aVL + S-wave on V3> 20 mm in women, or >28 mm in males); or b) the voltage criterion of Sokolow-Lyon (sum of the S-wave on V1+ R-wave on leads V5 or V6>38 mm); or c) the patient history specified the presence of ECG-LVH based on any other criterion. Kidney damage was evaluated by conventional laboratory tests. GRF was calculated automatically from serum creatinine using the simplified Modification of Diet in Renal disease (MDRD) equation [17]. Urine testing was also carried out to calculate the UAE, by the albumin/creatinine (A/C) ratio or the 24-hour UAE. Kidney damage was considered if: a) the calculated GFR was <60 ml/min/1.73 m^2^; or b) the patient presented an A/C ratio of ≥ 3.5 mg/mmol (30 mg/g); or c) the patient presented an UAE ≥ 30 mg/24 hours.

The presence of established CVD was defined according to the patient's clinical records, and included myocardial infarction, angina, heart failure, peripheral vascular disease and stroke.

### Statistical analysis

The sample size was calculated according to the main objective of the study and based on the expected prevalence of heart and kidney damage. For an expected prevalence of <10%, a sample size of 2401 hypertensive diabetic patients was estimated for a 95% confidence interval (CI) and an error of 1.2%. The sample was increased 4% to cover data losses, yielding a definitive size of 2500 patients.

Qualitative variables are shown with their frequency distribution. Quantitative variables are summarized by their mean, standard deviation (SD), range and percentiles. Asymmetric variables were described by the median and interquartile range (p25–p75). Association between qualitative variables was evaluated using the chi-square or the Fisher exact tests. The behavior of quantitative variables was analyzed for each of the independent variables using the Mann-Whitney U-test or median test.

A multivariable logistic regression model was made to account for the association of the study variables to the prevalence of established CVD. The odds ratios (OR) and corresponding 95% CI are presented.

Variable distribution was verified in all cases as compared with the theoretical models, and the hypothesis of homogeneity of variances was tested. In all hypothesis testing, the null hypothesis was rejected with a type I error or an alpha error <0.05. The SPSS 11.0 statistical package was used throughout.

## Results

### Descriptive data

Information was collected on 2466 patients, a total of 127(5.2%) being excluded from the analysis because they failed to meet some inclusion criterion or lacked some essential information. The final sample comprised 2339 patients (mean age 68.9 years [SD 10.8, range 55–98], 48.2% females). The mean body mass index (BMI) was 29.9 kg/m^2^(SD 7.8). 42.9% had obesity (BMI ≥ 30 kg/m^2^), and 11.6% were smokers. Some antecedent of CVD was recorded in 51.3% of the patients while 14.6% had a history of atrial fibrillation. The mean BP was 148.3/80.4 mmHg (SD 15.1/11.5), and only 15% showed BP < 130/80 mmHg. The characteristics of males and females are summarized in Table 1.

**Table 1:** Characteristics of the patients included in the RICARHD study.

		**Total**	**Males**	**Females**
		Mean (SD)	Mean (SD)	Mean (SD)

Age (years)*		68.7 (10.8)	67.1 (10.3)	70.3 (11.0)
BMI (kg/m^2^)		29.9 (7.8)	29.4 (9.2)	30.4 (5.8)
Abdominal perimeter (cm)*		100.4 (14.2)	102.2 (13.3)	98.4 (14.9)
Systolic blood pressure (mmHg)*		148.3 (15.1)	144.0 (17.9)	152.8 (21.6)
Diastolic blood pressure (mmHg)		80.4 (11.5)	80.0 (11.2)	80.8 (11.9)
		%	%	%
BMI (kg/m^2^) classification	<25	15.8%	14.9%	16,8%
	25–29.9	41.3%	47.3%	34,8%
	≥ 30	42.9%	37.8%	48,4%
Moderate-severe alcohol consumption*		10.0%	17.5%	1.8%
Smoking*		11.6%	17.3%	5.5%
Hypercholesterolemia		64.6%	64.2%	64.9%
BP control		15.0%	15.3%	14.6%
Glomerular filtrate <60 ml/min/1.73 m^2^*		45.1%	37.9%	52.7%
Left ventricle hypertrophy on ECG		22.9%	23.7%	22.2%
Established cardiovascular disease**, *		51.3%	55.1%	47.2%
Atrial fibrillation*		14.6%	12.5%	16.9%

*p < 0.05 for the difference between sexes. BMI: body mass index; ECG: electrocardiogram.

** Includes myocardial infarction, chest pain, heart failure, stroke and intermittent claudication

### Target organ damage

ECG-LVH was present in 22.9% of the patients, (22.2% of women and 23.7% of males, p = NS). GFR <60 ml/min/1.73 m^2 ^was documented in 45.1% (52.7% of women and 37.9% of males, p < 0.001). Information on UAE was available in 1887 patients – abnormal values (A/C ratio ≥ 3.5 mg/mmol [30 mg/g], or UAE ≥ 30 mg/24 hours) being recorded in 58.7% of the subjects (52.8% of women and 64.2% of males, p < 0.001).

### Cardiac and renal damage, and the prevalence of cardiovascular disease

**Figure 1:**
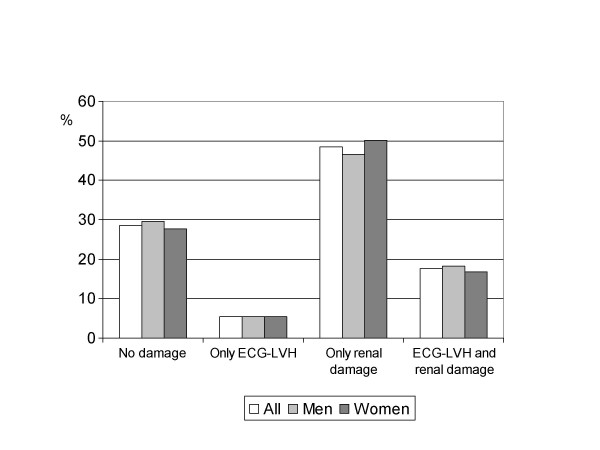
Distribution of the study population according to cardiac and/or renal damage. ECG-LVH: electrocardiographic left ventricular hypertrophy

To assess the relationship between the presence of cardiac and/or renal damage and of established CVD, the study population was divided into four groups: patients without cardiac or renal lesions (28.6%); patients with ECG-LVH but no kidney damage (5.4%); patients with kidney damage and no ECG-LVH (48.4%); and patients with both ECG-LVH and renal damage (17.6%). Figure 1 shows the frequency distributions for the global population and by gender.

The demographic characteristics of the four groups are shown in Table 2. Compared with the patients without ECG-LVH and without kidney damage, those with ECG-LVH showed (p < 0.05) higher mean systolic and diastolic BP values and a greater frequency of established CVD and atrial fibrillation. The patients with kidney damage were older, showed higher systolic BP and had a greater frequency of established CVD and atrial fibrillation (p < 0.05). Finally, those with heart and kidney damage were older, showed higher mean systolic BP and had a greater frequency of established CVD and atrial fibrillation, smoking and alcohol consumption, and hypercholesterolemia (p < 0.05).

**Table 2:** Characteristics according to the presence or absence of cardiac and renal damage.

	**No ECG-LVH and no renal damage** **(28.6%)** **(Group A)**	**ECG-LVH only** **(5.4%)** **(Group B)**	**Renal damage only** **(48.4%)** **(Group C)**	**ECG-LVH and renal damage** **(17.6%)** **(Group D)**
	Mean (SD)	Mean (SD)	Mean (SD)	Mean (SD)

Age (years)	66.2(10.6)	67.4(12.6)	69.8(10.6)^A^	69.9(10.3)^A^
Systolic blood pressure (mmHg)	142.9(17.4)	149.8(21.7)	151.1(21.2)	148.1(19.3)
Diastolic blood pressure (mmHg)	80.6(10.4)	83.1(12.1)^C^	79.6(11.8)	81.3(12.5)
BMI (kg/m^2^)	29.9	30.2	29.7	29.6
	%	%	%	%
Females	46.5%	48.0%	50.1%	46.1%
BMI ≥ 30 kg/m^2^	41.6%	48.8%	43.4%	41.9%
Moderate-severe alcohol consumption	9.8%	9.7%	8.2%	15.0%^A, B, C, D^
Smoking	10.3%	10.5%	10.5%	16.0%^A, B, C, D^
Hypercholesterolemia	62.1%	66.4%^A^	64.7%	70.0%^A, C^
BP control	14.2%	13.8%	17.0%^D^	11.2%
Established cardiovascular disease*	37.7%	58.9%^A^	51.4%^A^	70.6%^A, C^
Atrial fibrillation	7.8%	18.0%^A^	15.3%^A^	22.5%^A, C^

See text for significance. BMI: body mass index; BP: blood pressure. *Includes myocardial infarction, angina, heart failure, stroke and peripheral vascular disease. ECG-LVH: electrocardiographic left ventricular hypertrophy. Superscripts ^(A, B, C, D) ^mean p < 0.05 compared to the indicated group.

The prevalence of the different manifestations of CVD, stratified by the presence of absence of ECG-LVH and/or kidney damage, is reported in Table 3. Compared with the patients with no cardiac or kidney damage, the subjects with kidney or cardiac damage showed a higher prevalence of any type of CVD (p < 0.001). The highest prevalence of any CVD was present in those with both ECG-LVH and kidney damage.

**Table 3:** Prevalence of established cardiovascular disease according to the presence of cardiac and/or renal damage.

	**No ECG-LVH and no renal damage** **(28.6%)** **(Group A)**	**ECG-LVH only** **(5.4%)** **(Group B)**	**Renal damage only** **(48.4%)** **(Group C)**	**ECG-LVH and renal damage** **(17.6%)** **(Group D)**
Cardiovascular disease (any), %	37.7%	58.9%^A^	51.4%^A^	70.6%^A, C^
Angina, %	12.4%	19.7%^A^	14.7%	22.0%^A, C^
Myocardial infarction, %	13.1%	25.4%	17.5%	28.2%^A, C^
Heart failure, %	10.3%	23.8%^A^	20.6%^A^	33.8%^A^
Peripheral vascular disease, %	6.1%	10.8%	14.9%^A^	28.0%^A, B, C^
Stroke, %	11.9%	13.8%	18.2%^A^	23.7%^A^
Atrial fibrillation, %	7.8%	18.0%^A^	15.3%^A^	22.5%^A, C^

ECG-LVH: electrocardiographic left ventricular hypertrophy. Superscripts ^(A, B, C, D) ^mean p < 0.05 compared to the indicated group.

### Multivariate analysis

To evaluate the impact of ECG-LVH and kidney damage upon the prevalence of established CVD, a multivariate model was constructed that included age, sex, BMI, moderate-severe alcohol consumption, smoking, hypercholesterolemia and BP.

Compared with the reference patients (no ECG-LVH and no kidney damage), the presence of ECG-LVH was associated with a 2-fold increased prevalence of established CVD (adjusted OR 2.20, 95%CI 1.43–3.38), while the presence of kidney damage alone was associated with a 41% greater prevalence (adjusted OR 1.41, 95%CI 1.13–1.75). The concomitant presence of both, ECG-LVH and renal damage was associated with a 3-fold greater prevalence (adjusted OR 3.12, 95%CI 2.33–4.19) of established CVD. This relationship was shown for the whole population, and also after stratifying by gender (table 4).

**Table 4:** Odds ratio of prevalence of cardiovascular disease, related to cardiac and/or renal damage.

	**OR (95%CI) for cardiovascular disease**		
	
	Global*	**Male**	**Female**
No ECG-LVH or kidney damage (reference)**	1	1	1
Only ECG-LVH	2.20 (1.43–3.38) p < 0.001	2.06 (1.13–3.78) p = 0.018	2.39 (1.29–4.44) p = 0.006
Only kidney damage	1.41 (1.13–1.75) p = 0.002	1.61 (1.19–2.18) p = 0.002	1.27 (0.92–1.75) p = 0.148
ECG-LVH and kidney damage	3.12 (2.33–4.19) p < 0.001	2.92 (1.95–4.39) p < 0.001	3.50 (2.28–5.37) p < 0.001

Adjusted for age, body mass index, sedentarism, hypercholesterolemia, blood pressure control, smoking, alcohol consumption, atrial fibrillation and *sex. OR: odds ratio; ECG-LVH: electrocardiographic left ventricular hypertrophy. **Reference group.

## Discussion

The main findings of the RICARHD study were the following: 1) The presence of target organ damage (ECG-LVH, renal dysfunction, or abnormal UAE) is frequent in this group of patients with HT and type 2 DM seen in specialized clinics; 2) Such lesions are related to an increased prevalence of established CVD; and 3) The concomitant presence of cardiac and renal damage is associated with an even higher prevalence of cardiovascular complications. Thus, the integral evaluation of both types of lesion affords complementary information. The study was carried out in Internal Medicine and Nephrology outpatient clinics, and the conclusions drawn are applicable to the profile of the patients seen in such settings.

The prevalence of ECG-LVH was nearly 23%, based on simple voltage criteria, and was similar in both males and females. This prevalence may be slightly greater than expected, since the study was conducted in specialized centers. In other study conducted in the out-hospital setting in patients with DM (85% with concomitant HT), the prevalence of ECG-LVH based on the Cornell product was 17.1% [18]. Echocardiographic studies in turn report prevalences between 43% in the study of Sato et al. in patients with normal UAE not taking antihypertensive treatment [19] and 71% in the study of Dawson et al., conducted in the hospital setting [20].

In any case, LVH tends to be more prevalent in hypertensive diabetic patients than in non-diabetics [13], as is the case in patients with the metabolic syndrome [21]. Metabolic anomalies involving insulin resistance and hyperinsulinemia could favor the appearance of LVH independently of HT. At experimental level, insulin exerts trophic effects in animal models [22], while a number of human studies have reported a relationship among high insulin levels, insulin resistance and left ventricle mass [23-25]. In addition, insulin induces sodium retention at kidney level [26], which may also contribute to the development of LVH.

A total of 45.1% of the patients showed GFR <60 ml/min/1.73 m^2^. GFR was calculated using the simplified MDRD equation [17]. Although this equation may underestimate GFR by up to 29% in healthy subjects, this figure drops to only 6% in patients with genuinely reduced GFR [27], and is moreover the most widely used equation to calculate GRF. GFR decreases with increasing age, and the percentage of patients with diminished GFR recorded in our series is not surprising, moreover considering that HT and DM are independent risk factors for renal derangement. The greater prevalence of impaired kidney function among women has already been reported in other studies in our setting [28] and in other countries [29,30], and is a consequence of the correction included in the equation for the decrease in muscle mass in women.

The prevalence of pathological UAE was very high in our series (58.7%). This may be conditioned by the fact that some patients were evaluated in Nephrology clinics. Microalbuminuria is predictive of posterior impaired renal function [31], and cross-sectional studies also have revealed an independent relationship between insulin resistance and microalbuminuria [32]. The relationship between diminished GFR and the risk of cardiovascular complications and death has also been observed in different follow-up studies [7,8].

The most useful finding in our study was the relationship between silent target organ damage and established CVD. The prevalence of established CVD was twice as great in patients with ECG-LVH of either sex, between 30–60% greater in patients with kidney damage versus patients without ECG-LVH or kidney damage, and ever greater (three-fold) in those with both kidney and cardiac damage. This suggests that careful evaluation of these organs can improve patient risk assessment, and that the presence of kidney damage adds information to the presence of ECG-LVH and vice versa.

The data afforded by follow-up surveys and by cross-sectional studies thus support the need for correct assessment of damage to both target organs in patients with HT and DM, in order to define the cardiovascular risk and management strategy. The 2003 European Society of Cardiology/European Society of Hypertension Guidelines for the Management of Arterial Hypertension consider both, diabetes mellitus and target organ damage, as situations associated to a high 10-year cardiovascular risk (20–30%) even in subjects with high-normal BP [33]. This and other studies as the LIFE diabetes substudy address the question of if these diabetic hypertensive patients with target organ damage should fall into the very-high risk category (estimated ten-year risk of CVD over 30%). In the LIFE study, the mortality rate after 4.8 years of follow-up (half the follow-up that that used for the estimation in the Guidelines) was 14% for the subgroup of diabetic patients with HT and ECG-LVH [9], and 20,3% suffered a cardiovascular complication (cardiovascular death, myocardial infarction or stroke). Moreover, the higher risk was for those with UAE >16.9 mg/mmol (approximately equivalent to 150 mg/g), in whom mortality after 4.8 years was nearly 20%, and the incidence of cardiovascular complications was 26.4% [10].

In diabetic patients a BP control target of under 130/80 mmHg is accepted [33-35], and blocking of the rennin-angiotensin system is recommendable when kidney damage or ECG-LVH is detected. Different studies have shown that not only is such organ damage predictive of cardiovascular complications, but – more importantly – the regression of such lesions reduces the incidence of cardiovascular complications over the middle term. In the 1195 patients with HT, DM and ECG-LVH of the LIFE DM substudy [9], treatment with losartan (plus hydrochlorothiazide in most cases) reduced mortality by 40% versus treatment with atenolol (plus hydrochlorothiazide in most cases) and the greater reduction of microalbuminuria after one year of treatment was related to posterior reduction of the cardiovascular complications and in mortality [36]. A number of studies have also shown that in patients with HT and LVH, hypertrophy regression as demonstrated by both ECG [37,38] and echocardiography [39,40] is associated with an improved cardiovascular prognosis and that, moreover, the regression of both disorders (microalbuminuria and LVH) may improve the prognosis even more than regression of only one of the lesions [41]. Therefore, therapy in these patients should aim not only to control BP but also to induce regression of LVH and to reduce UAE.

Our study presents two major limitations: its cross-sectional nature and the setting in which it was carried out. The cross-sectional design only allows us to establish associations, without reliably defining the underlying cause-effect relationship. The important of some cardiovascular diseases involving high mortality, such as stroke, may be underestimated. The selection of Internal Medicine and Nephrology clinics for conducting the study means that the observed prevalences do not reflect the global population of hypertensive patients with type 2 DM. In fact, the prevalences of established CVD in this sample, as well as of renal damage, were extremely high. These high prevalences may be due not only to the setting in which the study was conducted but also because the selection of patients was not done at random: they were consecutively included, and this could have favored the inclusion of more sick patients (patients with established CVD), because they are usually more closely followed-up and attend the outpatient clinics more frequently. In this sense, the results of our study should apply only to this population and not to the universe of hypertensive type 2 diabetic patients. Nevertheless, the conclusions drawn in terms of the relationship between target organ damage and CVD are valid, and the size of the sample and the multicenter nature of the study offer a very reliable assessment of the population seen by such specialists.

## Conclusion

In conclusion, the prevalence ECG-LVH and of renal damage, diagnosed by simple methods, in this population of hypertensive patients with type 2 DM, is high, and is associated with an increased prevalence of established CVD. Moreover, each lesion is independently related to CVD – the simultaneous presence of both lesions affording complementary information. The methods used to evaluate these lesions are very simple and inexpensive, and their careful application may help to improve the evaluation and to establish therapeutic objectives and strategies in these patients with such important cardiovascular risk.

## Abbreviations

CVD: cardiovascular disease

HT: hypertension

DM: diabetes mellitus

LVH: left ventricular hypertrophy

ECG: electrocardiogram

ECG-LVH: electrocardiographic left ventricular hypertrophy

GFR: glomerular filtration rate

UAE: urinary albumin excretion

OR: odds ratio

CI: confidence interval

SD: standard deviation

BP: Blood pressure

MDRD: Modification of Diet in Renal disease

BMI: body mass index

A/C ratio: albumin/creatinine ratio

## Competing interests

LC is a full-time employee at the Clinic Research Department of Merk Sharp & Dohme, Spain. PC and PG have received fees from Merck Sharp & Dohme, Spain for lectures during the past five years. CF has received fees from Merck Sharp & Dohme, Spain as a consultant in statistics. FA does not declare any competing interest.

## Authors' contributions

LC, PC, PG anf FA conceived the study, and participated in its design. CF designed and made the statistical analysis. LC drafted the manuscript, and all authors reviewed and approved the final manuscript.
